# Factors Associated With Willingness to Share Health Information: Rapid Review

**DOI:** 10.2196/20702

**Published:** 2022-02-09

**Authors:** Iffat Naeem, Hude Quan, Shaminder Singh, Nashit Chowdhury, Mohammad Chowdhury, Vineet Saini, Turin TC

**Affiliations:** 1 O'Brien Institute of Public Health University of Calgary Calgary, AB Canada; 2 Department of Community Health Sciences Cumming School of Medicine University of Calgary Calgary, AB Canada; 3 School of Nursing and Midwifery Faculty of Health, Community and Education Mount Royal University Calgary, AB Canada; 4 Department of Psychiatry Cumming School of Medicine University of Calgary Calgary, AB Canada; 5 Research and Innovation – Provincial Population and Public Health Alberta Health Services Calgary, AB Canada; 6 Department of Family Medicine Cumming School of Medicine University of Calgary Calgary, AB Canada

**Keywords:** health information, information sharing, health data, EMR, PHR, mobile phone

## Abstract

**Background:**

To expand research and strategies to prevent disease, comprehensive and real-time data are essential. Health data are increasingly available from platforms such as pharmaceuticals, genomics, health care imaging, medical procedures, wearable devices, and internet activity. Further, health data are integrated with an individual’s sociodemographic information, medical conditions, genetics, treatments, and health care. Ultimately, health information generation and flow are controlled by the patient or participant; however, there is a lack of understanding about the factors that influence willingness to share health information. A synthesis of the current literature on the multifactorial nature of health information sharing preferences is required to understand health information exchange.

**Objective:**

The objectives of this review are to identify peer-reviewed literature that reported factors associated with health information sharing and to organize factors into cohesive themes and present a narrative synthesis of factors related to willingness to share health information.

**Methods:**

This review uses a rapid review methodology to gather literature regarding willingness to share health information within the context of eHealth, which includes electronic health records, personal health records, mobile health information, general health information, or information on social determinants of health. MEDLINE and Google Scholar were searched using keywords such as *electronic health records* AND *data sharing* OR *sharing preference* OR *willingness to share*. The search was limited to any population that excluded health care workers or practitioners, and the participants aged ≥18 years within the US or Canadian context. The data abstraction process using thematic analysis where any factors associated with sharing health information were highlighted and coded inductively within each article. On the basis of shared meaning, the coded factors were collated into major themes.

**Results:**

A total of 26 research articles met our inclusion criteria and were included in the qualitative analysis. The inductive thematic coding process revealed multiple major themes related to sharing health information.

**Conclusions:**

This review emphasized the importance of data generators’ viewpoints and the complex systems of factors that shape their decision to share health information. The themes explored in this study emphasize the importance of trust at multiple levels to develop effective information exchange partnerships. In the case of improving precision health care, addressing the factors presented here that influence willingness to share information can improve sharing capacity for individuals and allow researchers to reorient their methods to address hesitation in sharing health information.

## Introduction

### Background

In the age of precision medicine and precision public health, good-quality data are an imperative first step to inform clinical guidelines, best practices, and policies. Precision medicine focuses on individualized patient care, taking into account the variability in genes, environment, and lifestyle [1]. Precision public health emphasizes targeted intervention programs for disease prevention and health promotion to reduce health disparities in populations [2]. This is done by applying emerging methodologies in epidemiology, biostatistics, and computing systems, including machine learning and artificial intelligence. These concepts often intersect, where clinical guidelines developed from population-level studies are adjusted to an individual patient based on their unique characteristics, leading to optimal care [3].

To expand research, and medical and disease prevention agendas through the use of precision medicine and public health frameworks, comprehensive, real-time, and integrated data need to be available. A growing number of diverse data sources provide rich and complex information, including pharmaceuticals, genomics, health care imaging, medical procedures, wearable devices, and internet activity [4]. The potential to harness these data to inform health care systems and health delivery is vast. This can include research on large, shared medical data sets or population-level sources to formulate disease risk models for use at the point-of-care level and to inform precision policy [5]. Further, eHealth data are increasingly available and provide integrated information about an individual’s sociodemographic information, medical conditions, genetics, treatments, and health care use [6]. Collection, aggregation, analysis, and dissemination of electronically stored individualized health information allow for an opportunity to have a more integrated and coordinated health care system [7].

Multiple forms of health information exchange can occur: (1) health information sharing between health agencies, (2) individuals sharing health data with medical care providers, and (3) individuals sharing health data in health research studies, health social networks, biobanks, and nationwide health information exchanges [8]. Ultimately, the patient or participant is at the center of this information exchange network; therefore, understanding the willingness, interest, and motivation to provide health information is an important aspect that must be explored [9]. Willingness to share information pertains to the *intention* to perform the sharing behavior and can be defined as the extent to which a person is ready to share their intellectual capital with other individuals. Willingness may be viewed as a mediator between the factors that influence sharing health information, which make up a sharer’s cognitive thought process, and the act of sharing. The concept of the intention (or willingness) to share precedes the sharing behavior following the theory of planned behavior, as posited by Madden et al [10]. This theoretical framework outlines that the attitudes, subjective norms, and the perceptions of control a person has, influences the intention to perform a behavior. In the case of willingness to share health information, willingness to share may be dependent on the perception of that individual regarding how favorable or unfavorable the result of sharing would be [11]. In this case, willingness to share health information may be a careful weighing of factors that may operate as positive or negative to influence a person to contribute their information.

National surveys and eHealth information platforms can provide excellent opportunities to collect health information for research and surveillance but can only be done if they are shared by the individuals being questioned. Previous studies have reported a high proportion of participants willing to share their health information for multiple purposes (care improvement, research, or surveillance) [8,12,13]. Privacy concerns and the type of information shared are considered important factors in studies understanding sharing preferences among patients sharing information toward electronic health records (EHRs) or personal health records. However, sharing of health information is nuanced by the influence of multiple factors, which can include information security, uncertainty about the end use of information, altruism, personality traits, illness histories, and other attributes related to the context around information sharing [14].

To our knowledge, there is no synthesis of studies that summarize the factors that individuals consider when sharing their health information. A synthesis of the current literature might help us to be able to link the various correlates of health information sharing preferences to ultimately increase the data sharing potential in certain populations. This rapid review offers an alternative form of knowledge synthesis compared with systematic review, where the process of review conduction is simplified and result synthesis can be done in a timely fashion. The results of the rapid review are usually descriptive and provide readily available knowledge about a topic in order to inform further investigation and decision-making [15]. For the purposes of this study, conducting a rapid review is an essential first step in the understanding and conceptualization of the literature-reported factors associated with willingness to share health information. Further well-informed inquiries are possible only with this conceptualization.

### Objectives

Specifically, the objectives of this review are as follows:

1. To identify peer-reviewed literature that reported factors associated with health information sharing
2. To organize factors into cohesive themes and present the synthesis as factors related to willingness to share health information

## Methods

### Search Strategy

A search was conducted in MEDLINE (2008-2019) to gather literature regarding willingness to share health information within the context of eHealth, which includes EHRs, personal health records, mobile health (mHealth) information, general health information, and information on social determinants of health. Additional records were also identified using Google Scholar. The search keywords included *electronic health records* AND *data sharing* OR *sharing preference* OR *willingness to share* OR *health information sharing*. The search was limited to any population that excluded health care workers or practitioners, and the participants aged ≥18 years within the US or Canadian context (see Multimedia Appendix 1 for the complete search strategy).

### Identification of Records

One reviewer (IN) conducted an initial screening of the title and abstract, which identified records that were within the US and Canadian contexts and were limited to the primary peer-reviewed journal articles. Reviews, editorials, and commentaries were excluded. The screening was conducted in Excel (Microsoft Office 365; Microsoft Corp). Any ambiguous records were included for full-text screening. All included records from the title and abstract screening were saved in PDF format, and full text was screened by a single reviewer (IN). Studies were included if they reported a population aged >18 years, were not health care professionals, and reported on factors associated with sharing health information electronically or otherwise. Any ambiguity of full-text inclusion was resolved through discussion with the research team.

### Synthesis

The final record listed was imported to NVivo (version 12; QSR International) for data abstraction, which was done by a single researcher (IN). This process included an aspect of thematic analysis where any factors associated with sharing health information were highlighted and coded inductively within each article. This process was carried out by a single extractor (IN). This resulted in an extensive list of factors that were distinct, overlapping, or related. Through discussion with the research team about the interrelated nature of the factors, a consensus was achieved where the factors were collated into major themes. Additional information about each record was abstracted using a predesigned Excel spreadsheet form (Microsoft Office 365; Microsoft Corporation). The extracted information included study author, publication date, study type, main objectives, population, sample size, the type of health information discussed, and major conclusions. We present a narrative synthesis on factors related to health information sharing in this report.

## Results

### Overview

**Figure 1:**
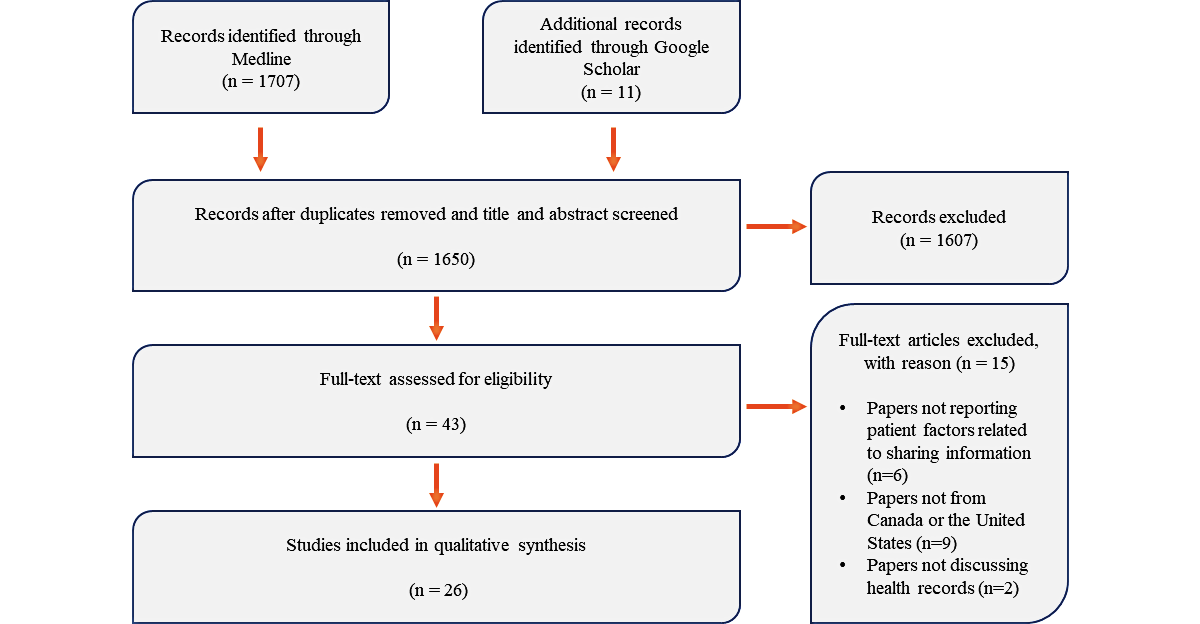
Study flow diagram.

The search was completed in October 2019. Initially, a total of 1707 records were identified through MEDLINE. Further, Google Scholar search yielded an additional 11 records for review. A total of 1650 unique records, after deduplication, were title and abstract screened, and thereafter, 1607 records were removed. Subsequently, 43 full-text articles were screened for relevance, of which 26 (60%) met the inclusion criteria and were analyzed for this review (Figure 1). The included studies in this paper reported on various populations using different methodologies. This included the general adult population using surveys, patient or hospital presenting populations (assessed using both survey and qualitative methods), and *other* groups of population, which included community-based studies or studies focusing on a particular population. The study characteristics are presented in Table 1.

**Table 1:** Summary of literature reviewed.

Study		Study type	Objective	Population	Sample size, N	Health information format discussed	Factors discussed influencing health data sharing	Major findings
**General adult population surveys**								
	Anderson and Agarwal [16]	Cross-sectional survey	Consumer willingness to provide access to patient health information to inform changes to policy	Adult public of the United States	1089	PHR^a^	Stakeholder use of health information Outcomes of health information Incentives to sharing health information	Contextual factors related to the requesting stakeholder and the purpose of information being requested influence patient trust on willingness to provide health information.
	Caine and Hanania [7]	Cross-sectional survey	A survey to understand patient preferences in sharing EMR^b^	Adult public receiving health care in the United States	30	EMR	Stakeholder use of health information Health information type and amount Patient engagement with health information Patient concern with data security and privacy Patient control over data	Participants were found to have preferences in type and amount of health information shared as a function of requesting stakeholders.
	Gaylin et al [17]	Cross-sectional interviews	To understand public attitudes regarding EMRs	General adult population of the United States	1014	EMR	Income and willingness to share health information Ethnicity and willingness to share health information Patients concern with data privacy and security	The overall public view of using EMRs in health care delivery are positive, and participants who had previous experience with IT^c^ are more likely to use and adopt EMRs.
	Cocosila and Archer [18]	Cross-sectional survey	To understand the consumer motivations to implement the used of PHRs by understanding individual barriers and motivators	Adult public in Canada	772	PHR	Stakeholder use of health information Mode of access to health information Age and willingness to share health information Engagement with IT and interest in PHR Patient engagement with health information Patient concern with data privacy and security	Participants with and without major illness are more likely to adopt and share electronic PHRs if they perceive it as useful and an advantage to themselves. Perceptions of data security, privacy, and trust are also important.
	Hasnain-Wynia et al [19]	Qualitative	To understand health plan members perceptions of the collection race, ethnicity, and primary language data	Health plan members in the United States	54	Health-related information	Ethnicity and willingness to share health information Patient engagement with health information Outcome of health information	Virtually no participants in the study had problem with discussing primary language, but participants had issues with sharing information regarding their ethnicity and race.
	Donovan-Kicken et al [20]	Cross-sectional survey	To explore factors related to health literacy in the comprehension and assessment of medical disclosure and consent forms	General adult population of the United States	254	Health-related information	Type and amount of health information shared	Health literacy and the comprehensible nature of consent documents for health research affect participation, especially with participant engagement with medical disclosure and consent documents.
	Kim et al [12]	Cross-sectional survey	To understand consumer characteristics, attitudes, and beliefs regarding consent to sharing eHealth data for health care and research purposes	General adult population of the United States	800	EHR^d^	Type and amount of health information shared Stakeholder use of health information Patient trust in researchers Health information for research Age and willingness to share health information Patient understanding of how data are used Patient control over data Patient concern with data security and privacy	Individual experiences and attitudes toward sharing of EHRs needs to be considered when using EHRs for research.
	Pickard and Swan [21]	Cross-sectional survey	To explore consumer attitudes toward sharing health information for research purposes	General population of the United States	128	Health-related information	Health information type and amount Stakeholder use of health information Patient understanding of how data are used Health data and management of disease Patient engagement with other patients Encouragement to share by stakeholders Patient control over data Patient concern with data security and privacy	Authors propose that health information sharing can be increased with trust, motivation, community, and informed consent.
	Medford-Davis et al [13]	Cross-sectional survey	To understand patient acceptability and benefit to sharing, consent to sharing, and benefit of health records	General population of the United States	1017	EHR	Health information type and amount Health information for research Patient understanding of how data are used Outcome of health information use Patient control over data Patient concern with security and privacy	Most participants of the study are in favor of HIE^e^ but would like more control of their health information through consent. Primary concerns with sharing health information includes concerns with privacy and security.
	Spooner et al [22]	Cross-sectional survey	To describe web-based health seeking behaviors and to identify patient-level factors to sharing of health information electronically with health care providers	General adult population of the United States	3677	Health-related information	Mode of access to health information Stakeholder use of health information Age and willingness to share health information	Participants of this study have high interest but low prevalence of HIE electronically.
	Weitzman et al [23]	Cross-sectional survey+qualitative	To investigate the willingness to share information contained in an EHR for use in public health monitoring and research	General population of the United States	181	EHR	Type and amount of health information shared Health information for research Patient understanding of how data are used Patient control over data Patient concern with data privacy and security	High levels of willingness were found in participants in sharing EHRs with public health for the purposes of disease monitoring, evaluation, and needs assessment, as guided by themes of altruism and pragmatism.
**Patient population or hospital presenting population—survey**								
	Bartlett et al [24]	Cross-sectional survey	To determine the factors that impact family medicine patients’ decision to allow their eHealth data to be used for research purposes	Attendees of family medicine clinics in Canada	474	eHealth data	Age and willingness to share health information Health information for research	Patients in family medicine clinics are more likely to refuse to contribute their deidentified eHealth data for research purposes. Relevance of the research to the patient was an impacting factor.
	Brown et al [25]	Survey	A survey study to understand the desirability and functionality of a communication portal in an ICU^f^	Adult ICU patients and family in the United States	2205	eHealth data	Stakeholder use of health information Mode of access to health information Age and willingness to share health information Patient engagement with health information	Current and potential ICU patients support the feasibility and effective information sharing facilitated by an eHealth information portal. Such a portal would help in providing clinical updates, documentation of family meetings, and information regarding health care staff roles.
	Garrido et al [26]	Retrospective observational study	To investigate the impact of race and ethnicity on PHR registration along with other factors	Adult members of health care network in the United States	1,764,121	PHR	Ethnicity and willingness to share health information	Racial groups of color were less likely to register for PHRs when controlling for other factors.
	Gerber et al [27]	Retrospective observational study	To understand the prevalence and patterns of PHR within an oncology population	Patents within a cancer center who had access to a secure web-based portal with their PHR in the United States	6495	EMR	Patient engagement with IT Ethnicity and willingness to share health information	Oncology patients readily adopt the use of EMRs. Explanatory factors are the greater health care need by these patients leads to increased portal use.
	Kerath et al [28]	Cross-sectional survey	To understand attitudes related to the collection, storing, and consent toward use of genetic information for research purposes	Long Island health system patients and their families	1041	Genomic data	Health information type and amount Patient trust in research Stakeholder requesting health information Age and willingness to share health information Patient understanding of how data are used Previous interaction with IT Patient concern with data security and privacy	Most participants were willing to share health information, where limitations to sharing were related to data privacy and consent procedures, along with importance of the studies being conducted.
	Padrez et al [29]	Cross-sectional survey	To explore the feasibility and data availability to linking patient’s social media content with their EMR data	Adult Facebook or Twitter users who presented to an emergency department	1433	EMR	Previous engagement with IT	Most individuals presenting to an emergency department that used social media consented to sharing and providing access to integrated information of their social media and EMR. The study presents a discussion on possible data repositories that link cross-platform data.
	Patel et al [30]	Cross-sectional survey	To explore consumer attitudes and support for physician use of HIE within a low-income, ethnically diverse community	Adult population presenting to an emergency and ambulatory care sites	214	PHR	Type and amount of health information shared Stakeholder use of health information Health information for research Age and willingness to share health information Health data and disease management Outcomes of health information use Patient concern with data security and privacy Patient control over health data	Over half of participants supported use of PHRs by themselves and their health care providers. Potential benefits of health information influences sharing.
	Pedersen et al [31]	Cross-sectional survey	To understand the acceptability of EHRs in an STI^g^ clinic and its impact on intention to be screened for STI	Patients of an STI clinic in Canada	1004	EHR	Type and amount of health information Stakeholder use of health information Age and willingness to share health information Patient concern with data security and privacy	One-third of participants reported that they were not comfortable with sharing their health information and are less likely to use STI clinic.
	Seltzer et al [8]	Cross-sectional survey	To explore participants willingness to share data, understand data content, and preferences related to sharing that data	Adult population presenting to an emergency department in the United States	206	Health-related information	Type and amount of health information shared Health information for research Patient understanding of how data are used Patient concern with security and privacy	Participants of the survey use a variety of modalities to generate data. Willingness to share health information for research increases for health-related insights.
	Teixeira et al [32]	Cross-sectional survey	To explore attitudes of patients with HIV about having their personal health information stored and shared electronically and what factors influence their willingness to share	Patients presenting to an HIV clinic in the United States	93	PHR	Stakeholder use of health information Ethnicity and willingness to share health information	Results indicate patients have a high trust in their primary care provider and HIV care teams and are willing to share information with these persons.
	Weitzman et al [33]	Cross-sectional survey	To investigate attitudes and practices related to sharing health information from an EHR to support patient care and public health monitoring	Patients or guardians who used EHRs in a hospital patient portal system	261	EHR	Type and amount of health information shared Stakeholder use of health information Age and willingness to share health information Patient understanding of how data are used Interest in PHRs Patient engagement with health information Patient control over data	The study found moderate levels of willingness to share electronically stored health information. Participants are more likely to share with public health authorities than are other stakeholders.
**Patient population or hospital presenting population—qualitative**								
	Courtney (2008) [34]	Qualitative	To understand concerns regarding willingness to adopt smartphone IT in senior citizens	Adults aged ≥65 years in residential care facilities in the United States	14	Smartphone IT information collection	Age and willingness to share health information Engagement with other information sharers or patients Patient concern with data privacy and security	Senior participants of this study indicate privacy as a barrier to the adoption of smartphone IT within their homes; however, their perceptions of the usefulness of the technology may be a mitigating factor.
	Fuji et al (2015) [35]	Qualitative	To understand the barriers and facilitators to sustained use of PHR in patients with type 2 diabetes in managing their disease	Adult patients with type 2 diabetes in the United States	59	PHR	Health data and management of disease Health data and management of disease Patient concern with data privacy and security Patient control over health information	Patients with type 2 diabetes experience multiple benefits of using PHRs, including disease management and facilitation of behavioral change. Sustained PHR use can be achieved via building strong patient-provider relationships.
**Other populations**								
	Beyer, et al [36]	Observational study	To explore the implications of having community engagement in the exploring and interpretation of a GIS^h^ disease mapping methodology for cancer	Rural community in the United States	60	GIS	Community engagement with health information Patient concern with data security	This study found that community interaction with GIS data for cancer was informative and allowed participants to build hypotheses and understanding of community health facilitating the ownership of their health data.
	Jamal et al [14]	Qualitative	To understand research participant attitudes toward confidentiality and data sharing of genomic information for research purposes	Adults who consented to genomic sequencing projects in the United States	30	Genomic data	Patient trust in researchers Health information for research Patient understanding of how data are used Outcomes of health information Patient concern with data security and privacy Patient control over data	A complex interplay of perception of data security and privacy, individual altruism, and situational collection and use of genomic information influences information sharing.

^a^PHR: personal health record.

^b^EMR: electronic medical record.

^c^IT: information technology.

^d^EHR: electronic health record.

^e^HIE: health information exchange.

^f^ICU: intensive care unit.

^g^STI: sexually transmitted disease.

^h^GIS: geographic information system.

**Figure 2:**
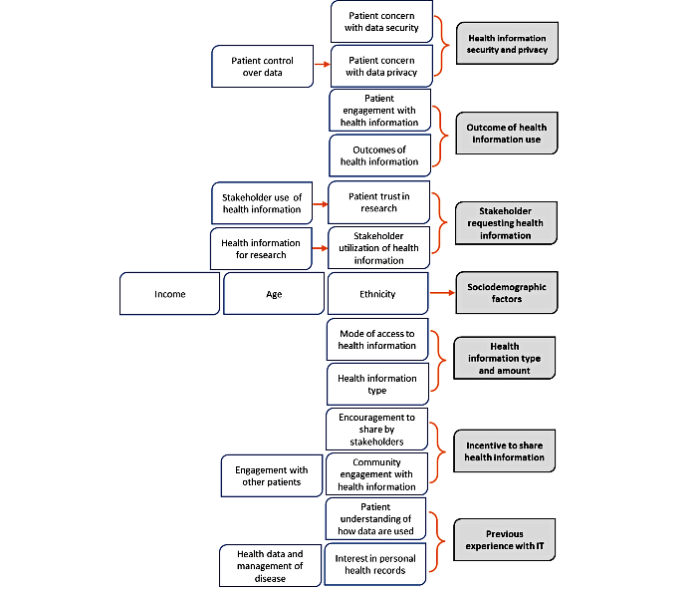
Factors related to willingness to share health information inductively coded within included papers and collated into major themes. IT: information technology.

The inductive thematic coding process revealed multiple factors related to willingness to share health information, as reported by the study participants (Figure 2). A single study often reported a multitude of factors related to sharing information (Table 1). The factors were collated into major themes related to sharing health information. For example, multiple studies reporting an association between age, income, or ethnicity, and the willingness to share health information were grouped under the major theme *sociodemographic factors*. A similar process was followed for the remaining factors coded within the research articles, from which 7 major themes emerged. The following is a narrative synthesis of all major themes discovered in the review process.

### Sociodemographic Factors

A total of 15 articles reported sociodemographic factors associated with willingness to share health information. The demographic factors as a major theme were primarily noted in the survey studies, in both the general adult population and a patient or hospital presenting population. The evidence suggests an incomplete exploration of the sociodemographic factors that operate in an interrelated manner to influence the willingness to share health information. For example, the relationship between age and willingness to share health information was contested, as some studies reported that older people were more comfortable with sharing health information because of a higher level of involvement with the health care system [25], whereas other studies found them to be less willing [18] as older users were less comfortable with information technology (IT) and, therefore, less likely to share health information, this being especially true via mobile phone apps. Others have found no influence of age (or other demographic variables) to be related to sharing health information for research purposes or to improve clinical care [22,28].

Measures of social capital have an unclear association with willingness to share health information also. Some papers have reported that higher education and income increase willingness to share health information and such individuals see the benefits of sharing information [22,24,33,37], whereas others have found no influence of these factors on sharing health information [12,28,31]. It should be noted that income and education are often covariates and their individual effects on outcomes are difficult to discern. Further, mediators, such as inequitable access to technology by lower socioeconomic groups, cannot be ignored when understanding willingness to share health data [23]. Further, although ethnic disparities have been noted regarding health information sharing [26], other researchers have found no effects of ethnicity and sharing health information [12,28,31].

### Incentive to Share Health Information

A total of 4 studies report the importance of incentives to increase willingness to share health information. Incentive in this case can be defined as something that drives and motivates individuals to perform an action. For the purpose of this analysis, the authors have considered incentives to be extrinsic (ie, financial) and intrinsic motivators. This theme was primarily presented in studies sampling from the general adult population and other populations, including a community [36] and individuals who consented to share their genomic data [14]. Individuals reported various incentives that may motivate them to share health information, including monetary and material incentives such as shopping credits or money [21].

Self-management of health as a result of health information sharing is another motivator of health information sharing, including the improvements in the understanding of the participants’ own health [37]. As individuals have an increased awareness of the ability to manage their health, this motivates people to share their health information. Health management can include actionable things such as knowing the likelihood of developing certain diseases, the current state of the person’s health, how health affects the social environments of the person, and receiving recommendations to improve health [8,21]. For example, the day-to-day management of health markers that some mHealth apps may offer (eg, physical activity tracking, blood pressure readings, and blood glucose readings) may be an incentive for users to be more engaged with the collection and sharing of health information [35]. Further, participants may be motivated to share health information if they could connect with other individuals who shared the same health conditions [21]. This is especially relevant to mHealth apps that offer engagement with a web-based community of users.

Finally, the reviewed studies also suggested that the public fundamentally cares about the purpose for which their information is being used and is more likely to share the information if it is being used for a good purpose [29]. Participants who perceived the outcomes and implications of their health information as useful were more likely to share their health information [14].

### Previous Experience With IT

A total of 12 research articles reported previous experience with IT as a factor associated with willingness to share health information. This theme was referenced by studies sampling from all types of populations, but especially so for survey studies assessing both the general and patient or hospital presenting populations. Respondents who showed interest and engagement in IT were more accepting of sharing health records [18,37]. Further, apprehension and anxiety perceived to using computers or wearables technology owing to lack of experience is a determinant of intention to share (eg, computer anxiety). Researchers argued for improving internet access and computer literacy as critical to increasing engagement and willingness to share health information, especially in a diverse population [37].

### Type and Amount of Health Information

A total of 14 studies reported factors related to the type and amount of health information in association with willingness to share health information. This theme was largely reported by survey studies, of both the general population and the patient or hospital presenting population. The results suggest that individuals prefer control over the type and amount of health data, where they can control the information being shared, with a primary concern being the confidentiality of their sensitive information [31]. This may include differing sharing practices based on the sensitivity of the information being shared (eg, sexual activity or orientation, adoptions, abortions, and substance abuse) [7]. The authors found that although most participants agreed with sharing their health information, they were less likely to be tested if participants knew that their clinical information was being shared by provincial health care systems.

### Data Privacy and Security

A total of 14 studies reported data privacy and security as a factor related to willingness to share health information. This theme was reported by all types of populations assessed. Within the growing trend of IT and the creation of large data repositories, security and privacy are a major concern for data producers and are closely linked to the confidentiality of sensitive information, as discussed in the previous section. Courtney [34] offers a multidimensional look into what privacy and security means within the health data field and found that patient mistrust results in withholding of health information. Fuji et al [35] found that privacy existed at both personal and technical levels, where some participants expressed themselves to be private and disliked sharing any information, whereas others stated that some technologies (eg, cloud sharing technology) may not be equipped to ensure total data security. Similar results in patients’ sensitivities to sharing health information have been found in genomics research [14,28].

In practice, although health information privacy and security are valued concepts for patients when sharing their EHRs, concerns about privacy decreased in specific patient groups, such as those who were chronically ill. In such cases, the benefits of sharing medical records may have outweighed privacy risks perceptions [18]. However, Gaylin et al [17] discussed the opposite, where privacy concerns were more important than sharing health information and its potential benefits to society. Further, mitigation of privacy concerns may increase willingness to share, such as anonymization [11,23]. However, researchers discussed that with the increases in IT systems to share information (eg, using social media), individuals may still be willing to share information regardless of privacy and security concerns.

### Stakeholder Requesting Health Information

Willingness to share health information is also influenced by who will use the information, which was as reported by 17 studies. This theme was primarily reported by survey studies (both general and patient or hospital presenting population). Studies showed that participants were more likely to share health information with their primary physicians, depending on the nature of the information [7]. Researchers and public organizations (nonclinical staff) were least likely to be on the list of participants’ willingness to share health information [7,11,32]. Hesitancy to share was especially true when the recipients of health information were doing research that was not relevant to the participants sharing information [24]. Participants were more likely to contribute information for research purposes if they knew that it would benefit themselves or the public in some way [14].

### Outcome of Health Information Use

A total of 10 studies reported that participants were influenced by the intended use and outcomes of their information when sharing health data. Again, this theme is mostly reported by survey studies, of both the general adult population and the patient or hospital presenting population. Anderson and Agarwal [16] found that the outcome and the role their health information had to play was important for sharing health information, as established trust was an important determinant of information sharing. Hasnain-Wynia et al [19] found that 90% of their study participants needed to know who was using their health information and for what purpose. Patel et al [37] found that individuals who perceived the positive benefits of sharing health information such as EHRs, such as understanding of their health, control over their health care, ability to make decisions together with their health care team, improvement in the quality of care, and satisfaction with health care, were more motivated to share their information. Brown et al [25] found that individuals who feel like they are contributing to an improvement of health care are more likely to share health information.

## Discussion

### Principal Findings

#### Overview

The purpose of this rapid review was to locate literature that reported factors related to willingness to share health information and synthesize them into cohesive themes. Through the review process, a total of 7 major themes were discovered that explored the different aspects of the process of sharing information. This included sociodemographic factors, contextual factors (eg, type and amount of health information shared), and a mix of contextual and cognitive factors that influence willingness to share (eg, stakeholder requesting information).

**Figure 3:**
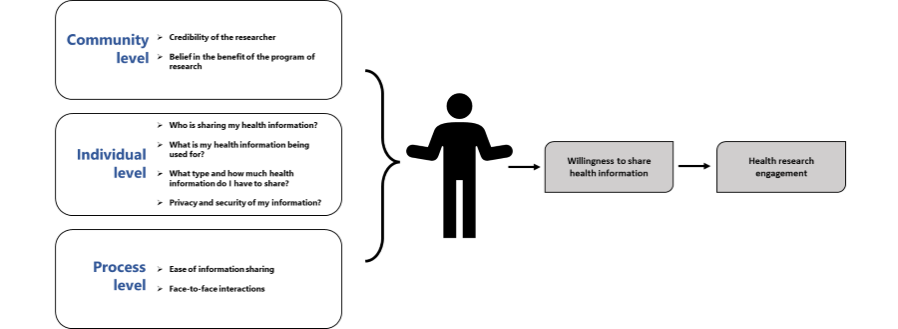
Dimensions of trust during the sharing process.

The factors associated with willingness to share health information reported here ultimately suggest the importance of developing trust. Trust is complicated, and often a philosophical concept, but is generally defined as imparting authority to another and accepting the vulnerability associated with that, given that a set of expectations are met [38]. When sharing their health information, an agreement of trust is made between the individual sharing and the stakeholder accepting the information. Participants share their information accepting that they have become vulnerable by sharing their intellectual capital and personal nature of health information, and rightly expect the outcome of that sharing process to meet their expectations. It is then up to the stakeholder to upkeep those expectations, or not, ultimately building or eroding that trust. Trust during the sharing process is multifaceted, and the factors associated with willingness to share health information that were found through this study illuminate some of these facets. When assessing the overlapping and unique themes found in this study, trust seems to operate at multiple levels: (1) community level, (2) individual level, and (3) process level (Figure 3).

#### Community-Level Trust

The first is community-level trust, which speaks to themes regarding the stakeholder requesting the information and the outcomes of information shared by participants in the studies reviewed within this report. Credibility in the institutions that back up the stakeholder is important, which was especially true if the institutions were well known and had a good reputation. The credibility aspect is particularly important for certain communities who have had historically less access to power and privilege and have been exploited in the name of health research. Credibility of the stakeholders can also mean that stakeholder appreciate the diversity within communities and are willing to engage with the community to understand their perspectives [39]. Further, the relatively less willingness to share health information that is sensitive in nature may be a universal aspect of sharing for all participants, but the compounding of historical research practices, mutual stereotypes, and differences in cultures and ethnicity can influence trust building between researchers and different types of communities.

Having knowledge about the purpose, benefits, and downsides to sharing their health information was also an important factor associated with willingness to share information. Understanding that sharing health information can benefit the participants individually or benefit the entire community builds resilience and contributes to the sense of community. A recent scoping review of barriers and facilitators of recruitment of South Asian participants found that engagement with health research was low in this population because of lack of knowledge about the scientific importance of the work, poor understanding of the research intentions, and the perception that the research benefits will not extend to their community [39].

#### Individual-Level Trust

The results of this study show that individual-level trust is built by a data-sharing environment where participants feel safe in sharing their health information. These factors evidently constitute a major decision-making aspect for participants when sharing health information. These factors include (1) stakeholder requesting information, (2) outcomes of health information, (3) security and privacy of health information, and (4) type and amount of health information shared. More importantly, the relative importance of these themes in this study may be because of their interrelatedness and connection with building individual-level trust through good research ethics.

The concept of data security and privacy of health data are well explored within the domain of health care, as health information is at times the most intimate, personal, and sensitive information that is maintained by the individual. Within most jurisdictions, privacy laws allow for total control over health information to the individuals, only to be disclosed if consent is authorized. Confidentiality goes a step beyond that and is usually characterized by an agreement between the individuals and the stakeholder requesting the information [40]. Indeed, participants felt that they would be more willing to share their health information if the information was going to be protected and private to a degree that they were comfortable with. Other studies have also found the sharing of information to be enhanced within the context of EHRs when privacy and security concerns were addressed [40,41].

Privacy and security of the data are closely linked to the outcome of that data, the stakeholder requesting the health information, and the type and amount of information shared. Participants are more likely to share their information if they feel they can have granular control over their shared data, which is also a form of maintaining privacy. If participants are able to control how much of their data and what type they are able to share, they have more control and feel safer in the sharing process. Participants also feel safe when they know the information is being used for its intended purpose, which is also communicated to them. For example, studies have shown the sociocultural aspects of collecting genetic information, which can be harmful or beneficial to the participants based on their familial and social circumstances [42].

Finally, who is using the health information is an important aspect of trust. Participants within the studies reviewed regularly stated they much preferred sharing their information with their physician or whomever primarily cared for them, health-wise. Studies have reported that individuals who regularly visit their physicians have a psychosocial expectation of benefit and trust from the physicians [43]. Having that interpersonal relationship built on the basis of day-to-day trust may be an important aspect to creating a space where health information sharing can occur. The lack of sharing of participants to other stakeholders, including organizations not associated with the health care of the participant, also points to the lack of trust and skepticism about the maintenance of privacy by these organizations.

#### Process-Level Trust

There is a paucity of literature describing the process of information sharing as having a role in participants’ willingness to share their information. Within populations, the ease of the information-sharing process can have a large influence on whether or not a participant will engage in sharing their health information. Factors as simple as language barriers, health literacy, and type of data collection instrument can determine a study’s success in engaging its population of interest [44]. In addition, complex factors, such as the sociodemographic diversity within a community, must also be addressed. For example, some ethnocultural communities may have first- and second-generation migrants who may have differing needs when it comes to ease of information sharing, where older-generation participants may require translation services or a different mode of data collection (face-to-face vs on the phone) to successfully share their information [45].

### Implications of Findings

The results of this study suggest actionable items that stakeholders can consider, including introducing policy changes that aim to develop a mutually beneficial information-sharing partnership between the communities of interest. Further, in order to motivate individuals to share their health information, their situations within their community must be appreciated, and equal power should be divided among the researcher and community members on the control and direction of the data-sharing partnership [46]. This is compared with researchers controlling the collection, analysis, and dissemination of the information along with reaping its benefits, with little input from participants. To build effective data-sharing partnerships, researchers should be able to work in collaboration with community members and understand the community living, working, and socializing conditions. To do this, credible and respectful access to the community should be pursued by building relationships with community champions and organizations that have a long-standing dedication to their communities. As suggested by the findings of this review, this can be done through training and development of guidelines that assist within building such relationships, which can exist at the institutional and national research level.

Another suggested actionable item could be the documentation of the process of rapport and building relationships with communities regarding building an information-sharing partnership, along with a systematic way of collecting the community perspectives on barriers and facilitators to sharing information. Although there is a great amount of literature using and describing methodologies that view research participants and the community as partners throughout the research process (eg, community-based participatory research and integrated knowledge translation), more exploration is required to create policies and guidelines for effective documentation of information-sharing partnerships.

A deeper understanding of conducting ethical research, the abstract nature of maintaining confidentiality, and respect for the individuals and their experiences is essential throughout the information-sharing process to develop trust. For example, many research studies suffer from the simplistic assumption that a single consent form is enough to assure ethical standards for their participants. However, the results of this study show that, within a community, more is needed. Indeed, a study can maintain excellent privacy and confidentiality within their protocol but may still conduct research that is framed in a way that is disrespectful toward certain ethnocultural communities [47]. Therefore, a reassessment of research ethics evaluation processes at the institutional levels may need to be improved and adjusted to address differences in conducting research in data-sharing communities.

Sharing of health information that is easy, accessible, and feasible for the participant can also cultivate trust. Having evidence-informed standards and clear guidelines for collecting health information can not only benefit stakeholders interested in gaining information by increasing reproducibility but also benefit the information-sharing partnership [48]. That being said, stakeholders should consider the population they are hoping to collect information from when choosing or creating these standards. For example, simply measuring the concept of ethnicity in populations can be difficult, as some participants may not see their ethnicity, or diversity within an ethnicity, being reflected in the type of questionnaire they are given. Further incentives are known to increase research engagement and may be an important aspect of building information-sharing partnerships in ethnocultural communities. However, simple financial incentives may not be enough to garner continued information sharing, but rather, more customized incentives may be needed for the communities that researchers are interested in. Studies have demonstrated that incentives for ethnic and minority communities, such as colearning activities and a chance to contribute to the research development, are sustainable incentives that build trusting partnerships [31].

### Limitations, Strengths, and Next Steps

This review is limited by its rapid review methodology, which fails to conduct a broader search of the literature and critically analyze the included studies. For example, this review contains studies with a variable sample size, which could influence the generalizability of the results of certain studies with smaller sample sizes. Further, the included studies report on the incomparable context of individuals, where some participants are hospitalized patients, as compared with the general adult population (Table 1). When assessing the results of this study, there are some notable differences in results when comparing the population assessed and the methods used to assess them. For example, some themes are overly represented in survey studies in both the general adult population and a patient or hospital presenting population simply because of the methods used. In survey studies, authors can quickly measure study participant preferences on the type and amount of health information shared, outcomes of health information use, and the stakeholder requesting health information. Further, most survey studies feature a larger population size, which can also influence the results by the inclusion of more viewpoints and more possible factors that influence willingness to share. However, the study finds its strengths in the reporting of concise narrative synthesis of factors associated with willingness to share health information into cohesive themes and subsequent domains, using thematic coding methods. An important next step for this review would be a systematic search of the literature, allowing for an in-depth analysis of health information sharing. Further, primary studies focusing on health information exchange in populations facing health disparities are warranted to expand the field.

### Conclusions

This review provided a concise report on factors associated with willingness to share health information, including a conceptual framework that outlined sociodemographic, cognitive, and contextual domains associated with health information sharing. Further, this review emphasized the importance of data generators’ viewpoints and the complex systems of factors that shape their decision to share health information. The factors related to information sharing reported in this review have important implications in participant engagement and reorientation of methodologies in research studies to build sustained information exchange capacity. Sustained information exchange is an important aspect of current trends in medical research and public health.

## Appendix

Multimedia Appendix 1 MEDLINE search strategy.

## Abbreviations

EHR: electronic health record
IT: information technology
mHealth: mobile health
