# Supplementary material for: Factors Associated With Willingness to Share Health Information: Rapid Review
Source: JMIR Hum Factors. 2022 Feb 9;9(1):e20702. doi: 10.2196/20702 (PMC8867291; doi:10.2196/20702)
Supplement: Multimedia Appendix 1 [file humanfactors_v9i1e20702_app1.docx]

**Multimedia Appendix 1.** MEDLINE search strategy.

| **#** | **Searches** | **Results** |
| --- | --- | --- |
| 1 | exp Data Collection/ | 2126668 |
| 2 | exp Medical Records Systems, Computerized/ or exp Electronic Health Records/ or exp Hospital Information Systems/ | 60682 |
| 3 | "data collection".ab,ti. | 72258 |
| 4 | EMR.ab,ti. | 6231 |
| 5 | EHR.ab,ti. | 6563 |
| 6 | exp Information Dissemination/ | 16219 |
| 7 | "electronic health record* ".ab,ti. | 14133 |
| 8 | "electronic medical record* ".ab,ti. | 15264 |
| 9 | "open data".ab,ti. | 764 |
| 10 | "health information sharing".ab,ti. | 61 |
| 11 | "health data sharing".ab,ti. | 42 |
| 12 | "data sharing".ab,ti. | 3400 |
| 13 | "health data".ab,ti. | 6462 |
| 14 | (sharing adj3 data).mp. [mp=title, abstract, original title, name of substance word, subject heading word, floating sub-heading word, keyword heading word, organism supplementary concept word, protocol supplementary concept word, rare disease supplementary concept word, unique identifier, synonyms] | 5699 |
| 15 | (sharing adj3 "health data").mp. [mp=title, abstract, original title, name of substance word, subject heading word, floating sub-heading word, keyword heading word, organism supplementary concept word, protocol supplementary concept word, rare disease supplementary concept word, unique identifier, synonyms] | 119 |
| 16 | (sharing adj3 "health information sharing").mp. [mp=title, abstract, original title, name of substance word, subject heading word, floating sub-heading word, keyword heading word, organism supplementary concept word, protocol supplementary concept word, rare disease supplementary concept word, unique identifier, synonyms] | 61 |
| 17 | exp Patient Preference/ | 8061 |
| 18 | exp Privacy/ | 15135 |
| 19 | exp Confidentiality/ | 52388 |
| 20 | "patient preference".ab,ti. | 3876 |
| 21 | (preference adj3 patient).mp. [mp=title, abstract, original title, name of substance word, subject heading word, floating sub-heading word, keyword heading word, organism supplementary concept word, protocol supplementary concept word, rare disease supplementary concept word, unique identifier, synonyms] | 12366 |
| 22 | privacy.ab,ti. | 14644 |
| 23 | confidentiality.ab,ti. | 10908 |
| 24 | exp Social Responsibility/ | 23579 |
| 25 | accountability.ab,ti. | 12449 |
| 26 | "social responsibility".ab,ti. | 1431 |
| 27 | (preference adj3 sharing).mp. [mp=title, abstract, original title, name of substance word, subject heading word, floating sub-heading word, keyword heading word, organism supplementary concept word, protocol supplementary concept word, rare disease supplementary concept word, unique identifier, synonyms] | 31 |
| 28 | "sharing preference".ab,ti. | 3 |
| 29 | exp Security Measures/ | 14215 |
| 30 | security.ab,ti. | 44536 |
| 31 | "willingness to share".ab,ti. | 199 |
| 32 | exp Health Personnel/ or students, health occupations/ or students, dental/ or students, medical/ or students, nursing/ or students, pharmacy/ or students, public health/ or Social Workers/ or (((acupuncturist* or allergists or anatomist* or anesthesiologist* or anesthetist* or audiologist* or cardiologist* or chiropractor* or clinican* or dental hygienist* or dentist* or dermatologist* or diabetologist* or dietician* or doctor* or doula or doulas or endocrinologist* or gastroenterologist* or general practitioner* or geriatrician* or gynecologist* or haematologist* or (health or health care or healthcare)) adj2 (worker* or practitioner* or provider or professional or navigator* or student*)) or hospitalist* or internist* or medical resident* or medical student* or midwife or midwives or neonatologist* or nephrologist* or neurologist* or neurosurgeon* or nurse or nurses or nursing student* or nutritionist* or obstetrician* or oncologist* or ophthalmologist* or optometrist* or osteopath or osteopaths or otolaryngologist* or pathologist* or pediatrician* or pharmacist* or pharmacolog* or phlebotomist* or physician* or podiatrist* or prosthetist* or psychologist* or psychiatrist* or pulmonologist* or radiographer or radiologist* or radiotherapist* or rheumatologist* or social worker* or sonographer* or surgeon* or therapist* or toxicologist* or urologist* or veterinarian*).mp. | 4879452 |
| 33 | exp Canada/ | 155406 |
| 34 | Canada.ab,ti. | 81758 |
| 35 | exp United States/ | 1329708 |
| 36 | USA.ab,ti. | 98966 |
| 37 | "United States of America".ab,ti. | 3609 |
| 38 | 2 or 4 or 5 or 7 or 8 or 9 or 13 | 86935 |
| 39 | 1 or 3 or 6 or 9 or 10 or 11 or 12 or 14 or 15 or 16 | 2192576 |
| 40 | 17 or 18 or 19 or 20 or 21 or 22 or 23 or 24 or 25 or 26 or 27 or 28 or 29 or 30 or 31 | 165373 |
| 41 | 38 and 39 and 40 | 6675 |
| 42 | 41 not 32 | 5146 |
| 43 | 33 or 34 | 191136 |
| 44 | 35 or 36 or 37 | 1404184 |
| 45 | 42 and 43 | 135 |
| 46 | 42 and 44 | 2088 |
| 47 | 45 or 46 | 2210 |
| 48 | limit 47 to (English language and yr="2008 -Current" and "all adult (19 plus years)") | 105 |
| 49 | 39 and 40 | 42748 |
| 50 | 49 not 32 | 29556 |
| 51 | 43 and 50 | 1075 |
| 52 | 44 and 50 | 8907 |
| 53 | 38 and 40 | 7949 |
| 54 | 53 not 32 | 6111 |
| 55 | 43 and 54 | 149 |
| 56 | 44 and 54 | 2448 |
| 57 | 51 or 52 or 55 or 56 | 10249 |
| 58 | limit 57 to (English language and yr="2008 -Current" and "all adult (19 plus years)") | 1707 |
| 59 | 48 or 58 | 1707 |
